# Supplementary material for: Comparative Microbial Profiles of Colonic Digesta between Ningxiang Pig and Large White Pig
Source: Animals (Basel). 2021 Jun 23;11(7):1862. doi: 10.3390/ani11071862 (PMC8300102; doi:10.3390/ani11071862)

Supplementary Information

Supplementary Table S1. Raw reads and selected effective sequences in each group.

Sample_name	Raw_reads(#)	Clean_Reads(#)	Base(nt)	AvgLen(nt)	Q20	GC%	Effective%
NX.1	65110	63875	26489375	414	80.83	52.69	98.1
NX.2	79336	74044	30886602	417	78.76	52.55	93.33
NX.3	63944	62607	25859956	413	79.95	52.53	97.91
NX.4	67123	61183	25221110	412	78.91	52.72	91.15
NX.5	84177	80265	33084136	412	79.25	52.78	95.35
NX.6	80016	76521	31639436	413	80.3	53.09	95.63
NX.7	68087	65771	27126396	412	80.32	52.84	96.6
NX.8	69678	63305	26227411	414	78.88	52.85	90.85
LW.1	75822	70272	29012688	412	80.18	52.94	92.68
LW.2	71438	70402	29020908	412	80.24	52.8	98.55
LW.3	92180	91066	37686652	413	83.77	53	98.79
LW.4	88905	84840	35271451	415	79.69	52.78	95.43
LW.5	70237	67238	28045529	417	78.81	52.62	95.73
LW.6	63747	60287	25142490	417	79.94	52.38	94.57
LW.7	69065	65522	27220430	415	80.45	52.79	94.87
LW.8	70412	67832	28049244	413	80.21	52.95	96.34

Table S2. Number of observed species, richness and diversity indices in the caecal samples from each dietary treatment.

Sample name	Observed_species	Shannon	Simpson	Chao1	ACE	Goods_coverage	PD_whole_tree
NX.1	718	7.026	0.981	753.253	758.244	0.998	51.48
NX.2	733	6.929	0.979	814.014	798.472	0.997	59.856
NX.3	541	6.356	0.973	605.556	593.965	0.998	43.375
NX.4	665	6.366	0.955	680.374	697.004	0.998	49.706
NX.5	696	6.603	0.965	820.744	802.956	0.996	49.315
NX.6	704	5.949	0.94	799.25	793.901	0.997	51.853
NX.7	714	6.441	0.957	768.529	782.097	0.997	52.644
NX.8	563	6.483	0.974	610.443	612.971	0.998	43.878
LW.1	634	6.306	0.954	682.95	689.891	0.998	47.926
LW.2	759	6.443	0.95	820.933	834.598	0.997	54.632
LW.3	695	6.379	0.955	766.959	759.72	0.997	49.88
LW.4	762	7.1	0.979	860.413	849.348	0.997	54.19
LW.5	719	6.912	0.978	788.709	790.814	0.997	52.9
LW.6	701	6.096	0.933	763.817	762.174	0.997	51.754
LW.7	690	6.757	0.971	733.076	738.697	0.998	51.244
LW.8	640	6.097	0.941	666.636	685.004	0.998	47.255

Figure S1 The top 35 genera were identified across all samples between two pig breeds

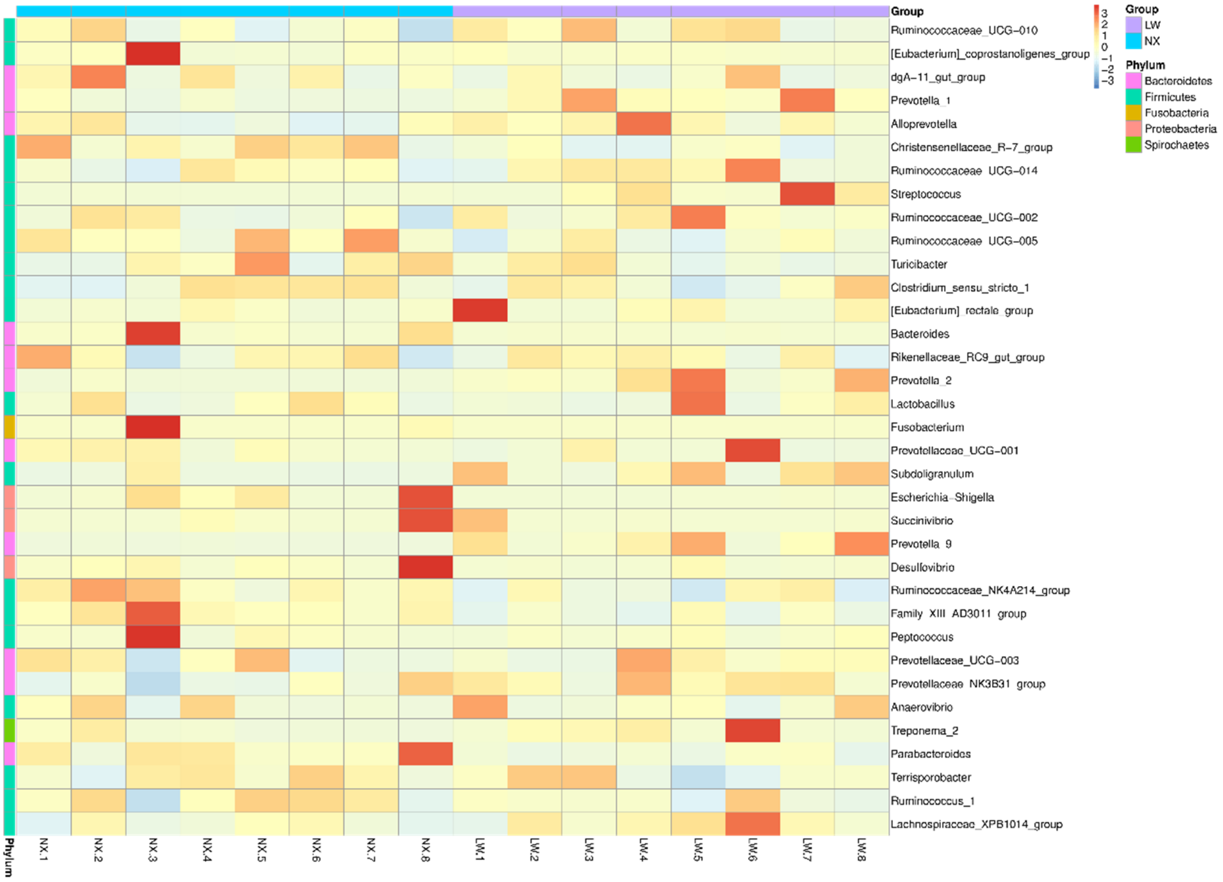

Supplement: Supplementary file 1 [file animals-11-01862-s001.zip › animals-1232895-supplementary.pdf]
